# Whom should we ask? A systematic literature review of the arguments regarding the most accurate source of information for valuation of health states

**DOI:** 10.1007/s11136-020-02426-4

**Published:** 2020-02-03

**Authors:** Gert Helgesson, Olivia Ernstsson, Mimmi Åström, Kristina Burström

**Affiliations:** 1 Stockholm Centre for Healthcare Ethics, Department of Learning, Informatics, Management and Ethics (LIME), Karolinska Institutet, 171 77 Stockholm, Sweden; 2 QRC KI, Department of Learning, Informatics, Management and Ethics (LIME), Karolinska Institutet, 171 77 Stockholm, Sweden; 3 Health Outcomes and Economic Evaluation Research Group, Stockholm Centre for Healthcare Ethics, Department of Learning, Informatics, Management and Ethics (LIME), Karolinska Institutet, 171 77 Stockholm, Sweden; 4 Equity and Health Policy Research Group, Department of Global Public Health, Karolinska Institutet, 171 77 Stockholm, Sweden; 5 Health Care Services, Region Stockholm, 171 77 Stockholm, Sweden

**Keywords:** Described health state, Experienced health state, General public values, Health state valuation, Hypothetical health state, Patient values

## Abstract

To determine and critically evaluate the arguments in the published literature regarding the most accurate source of information for valuation of health states: values based on experienced health states (patient values) or values based on described health states (general public values).

**Electronic supplementary material:**

The online version of this article (doi:10.1007/s11136-020-02426-4) contains supplementary material, which is available to authorized users.

## Introduction

Who should be asked to value health states, defined in terms of generic health dimensions, is an ongoing debate among economists, philosophers and other researchers in the field of health outcomes research [1–10]. There are two main approaches to eliciting health state valuations: individuals’ valuations based on their experience of the health state or individuals’ valuations based on a description of the health state. The choice of whose values to use for health state valuations has generally been described as a choice between ‘patient values’ versus ‘general public values,’ i.e., between those experiencing and those not experiencing the health state to be valued [7, 10]. The former has also been referred to as ‘individual values’ and the latter as ‘social’ or ‘hypothetical values’ [5, 11–15].

The need for clarification and interpretation of the terminology used in health state valuations has been raised [10, 16]. For instance, ‘experience-based values’ has been referred to as the value of the individual’s currently experienced health state [17]. However, experience might be based not only on current experience of the health state to be valued, but also on previous personal experience of the health state, experience of another health state similar to that to be valued, or experience based on relatives’ or other persons’ ill health [18]. When patients are asked to value health states, they are often valuing their own current health state, but patients may also value other health states described to them. Furthermore, the valuation process to elicit experience-based values might involve the imagined states ‘full health’ and ‘being dead.’ When the source of values is the general public, the values are hypothetical in the sense, and to the extent, that usually none of the health states to be valued is experienced by the individual. However, by chance, some of the respondents in the general public might currently experience or have experience of the health state to be valued. When patient values are used, then usually only one health state (their own) is valued by the respondent. When general public values are used, several health states are usually valued by the respondent.

In the literature, it has commonly been assumed that experience-based values must be obtained from patients (i.e., patients are the only kind of raters/respondents thought of as having experience of a health state) and that general public values must be values for described health states only. However, in some recent studies, the general public has been the source of values for valuing experience-based health states; i.e., the general public has been asked to value their own current health state [17, 19–21].

In economic evaluation of health technologies and health care interventions, the Quality-Adjusted Life Year (QALY), which combines length of life and health-related quality of life (HRQoL) into one measure, is commonly used [7]. In the QALY context, the quality of life component is usually based on preferences for health states. The Washington Panel on Cost-effectiveness in Health and Medicine recommended in 1996 that “for purposes of resource allocation, the relevant preferences are those of the general public” [22]. Such values, i.e., individuals’ valuations based on a description of the health state obtained from a sample of the general public, have been the most common. However, there have also been arguments in support of obtaining valuations based on individuals’ experience of the health state [4, 5, 19, 23–26].

The National Institute for Health and Care Excellence (NICE) in England and Wales [27] recommends the use of general public preferences, i.e., valuations of described states, in their guidelines for economic evaluations. However, in Sweden, the Dental and Pharmaceutical Benefits Agency (TLV) states that “QALY weightings based on appraisals of persons in the health condition in question are preferred before weightings calculated from an average of a population estimating a condition depicted for it (e.g., the ‘social tariff’ from the EQ-5D)” [28, 29].

Strengths and weaknesses with these main approaches to health state valuations have been discussed in the scientific literature, but to our knowledge, no systematic review of the arguments has so far been published. The aim of this paper was to determine and critically evaluate the arguments in the published literature regarding the most accurate source of information for valuation of health states: values based on experienced health states or described health states.

When critically evaluating arguments in the literature, we try to take as little as possible for granted when it comes to theoretical starting points. For instance, we leave it open whether the value of a health state should be understood primarily in terms of preferences for the state in comparison with other states, in terms of what it is like to be in the state in question, or some other option [3, 8, 9, 16, 30]. We are, furthermore, open also to the view that the value of a health state can be understood independently of the addition ‘according to whom,’ i.e., that the value of a health state is something intrinsic to that state rather than a relation between the state and one or another group of evaluators. Besides, we are aware that ‘value’ can be used to denote either the goodness/badness of the health state or the result of the valuation of the goodness/badness of the health state, which is something else.

In the methods section, we describe the search strategy, publication selection, data extraction, and process of synthesis. The two sets of arguments and our analysis of the data are presented in the results section. The paper ends with a discussion of the arguments and their relevance for the question regarding the most accurate source of information for valuation of health states.

It is outside the scope of this paper to address how the different approaches influence cost-utility ratios, as well as to address which method (such as time trade-off, standard gamble, or visual analogue scale) to use for valuation of health states. Also the paper does not rest on any elaborated views on how to understand ‘experience’ and ‘described’ health state, since that would go beyond the preciseness of most of the literature reviewed.

## Material and methods

This paper is based on a systematic review of arguments in the published literature regarding the most accurate source of information for valuation of health states. The two main approaches to eliciting health state valuations, values based on experienced health states and values based on described health states (hereafter labeled ‘patient values’ and ‘general public values,’ respectively), have been expressed in a variety of ways in the literature.

### Search strategy

A systematic search for relevant literature was conducted in October 2015 in three electronic databases: Scopus, Ovid Medline, and Econlit. The search was later updated to include publications until October 2017. The index terms were Medical Subject Headings (MeSH) and free text. The search strategy is presented in Online Appendix.

### Selection of publications

All identified publications were independently screened by two readers (second and third author). Potentially relevant publications were first identified through screening of *title and abstract*. They were considered relevant if they contained (1) health state valuations comparing patient values and general public values, or (2) a discussion of perspective when valuing health states. Publications were then read in *full text* if at least one of the reviewers considered it relevant for the review, applying the same inclusion criteria as above. In this preliminary reading of the full texts, publications were removed either because they were not written in English or because they were deemed as clearly not relevant for this paper (it was easily determined that they did not contain any arguments of the kind relevant for this paper).

**Fig. 1:**
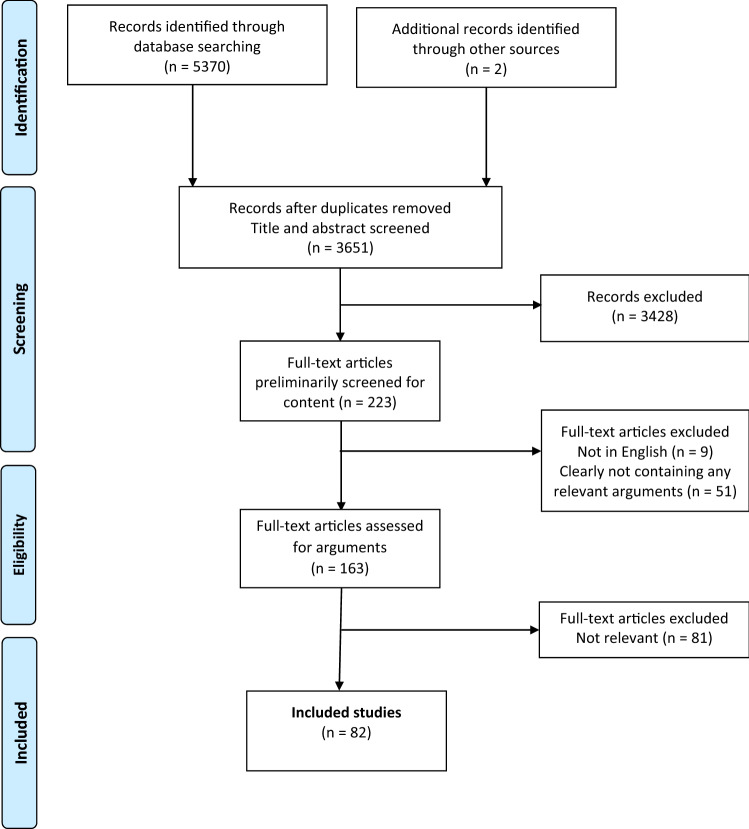
Flow chart on data search

As our aim of this paper was to determine and critically evaluate the *arguments regarding the most accurate source of information* for valuation of health states, when the remaining publications were read in full text after this preliminary screening, only those publications that contained arguments for one or the other of the two alternatives (using patient values or general public values) were included in the final analysis. For a flowchart on data search, see Fig. 1.

Our analysis did not concern arguments regarding choice of other sources for valuation of health states, such as arguments in favor of using valuations of politicians or medical experts.

### Data extraction and process of synthesis

A portion (*n* = 31) of the publications considered eligible for analysis (*n* = 163) was first read through independently by two readers (first and last author) in order to test the procedure of identifying arguments in favor of any of the two alternatives. Notes were taken according to a scheme where each argument in favor of one or the other of the two alternatives was stated schematically (“A is better than B since … [argument],” or vice versa, where “A” stood for general public values and “B” stood for patient values), exemplified by a quotation from the paper (with page and paragraph reference), and given a preliminary label (such as “The patients-are-better-informed argument”). The procedure was further tested at an early stage by including a third independent reader where all three readers read two papers and compared the outcomes in terms of identified arguments.

When the 31 selected publications had been read by the two readers, a full comparison of the lists of extracted arguments was made. When similar outcomes were noted, the readers discussed and agreed upon the best way to phrase the argument, the most adequate quotation to support it, and the best way to label the argument. Here existing labels from the literature were considered and used when found adequate. When different outcomes were noted—either that one reader had identified an argument that the other reader had not, or that there was disagreement about the content and point of a stated argument identified by both readers—then there was a discussion about what would be the most reasonable way to interpret the relevant passage. When agreement was reached, the best way to phrase the argument, the most adequate quotation, and the best label for it was discussed and agreed upon. It was agreed on beforehand that if agreement could not be reached regarding a certain presumed argument, then that argument would be disregarded (this did not in fact occur).

The rest of the publications (*n* = 132) considered eligible for analysis were divided between the two readers, who read them, discarded some of them as not containing any relevant arguments, and classified the arguments found as above, and consulted the other reader when needed.

### Synthesis

The outcome of this analysis (lists of arguments, sources for the arguments, and labels for arguments for each included publication) formed the basis for identification of first-, second-, and third-level themes, in accordance with common practice in descriptive qualitative content analysis [31, 32]. The grouping into themes was handled separately for the two approaches: using patient values or using general public values.

Preliminary first-level themes were identified by bringing together identical or very similarly stated arguments from the different publications where they occurred. This list of themes was discussed and successively shortened during the process of comparing the variation in expressions (“Is this the same argument or is it another argument?”). The purpose was to eliminate any duplications and overlaps, but without losing any distinct argument in the process. This operational process was complicated as the initial list of themes included variants for most of the themes. The reduced combined list formed the set of first-level themes.

In the next step, related first-level themes were distilled into second-level themes. This process was in some parts simple and straightforward, but in others a fairly complex and dynamic process where themes were arranged and re-arranged until the emerging second-level themes were seen as clearly distinct and adequately stated. The process of distilling second-level themes from first-level themes was first carried out independently by the two readers. Then, the two sets of suggestions for second-level themes were compared. The discrepancy between the suggestions was minor and consensus was reached through discussion. The third-level themes emerged from the readers’ individual examination of the second-level themes and a discussion to reach consensus (in one case). The first-, second-, and third-level themes were then critically discussed with the second and third author to get their input on the categorization and the labeling of the themes.

## Results

Through the database search, 3651 articles were identified after removing duplicates (Fig. 1). Of these, 223 articles were considered potentially relevant and the publications were read in full text. Nine articles were excluded as the full text was in another language than English, and 51 articles were considered not relevant as they did not meet the inclusion criteria. As a result of this full-text screening, 163 articles were left to be thoroughly assessed for arguments. Based on this assessment, another 81 articles were excluded as not relevant (i.e., not containing any arguments for either of the approaches). Finally, 82 articles were included because they contained arguments for either of the approaches.

The analysis of arguments favoring either values of experienced health states (patient values) or values of described health states (general public values) as a basis for valuation of health states resulted in two structured sets of arguments, one for each position. The analysis of arguments in favor of valuations based on patient values resulted in 16 first-level themes (Table 1), five second-level themes, and three third-level themes: ‘failures of the general public to value health states,’ ‘effects of measuring health states’ and ‘theoretical reasons’ (Table 2). The analysis of arguments in favor of valuations based on general public values resulted in 19 first-level themes (Table 3), five second-level themes, and three third-level themes: ‘patient failures to value health states,’ ‘ideas regarding how to reach the most acceptable societal outcome,’ and ‘possibilities and effects of measuring health states’ (Table 4). References to the articles included in the analysis are listed in Table 5 (see Online Appendix). Arguments to the effect that patient values are more accurate to a great extent concerned the idea that patients are better informed about the health state than the general public, circumstances regarding adaptation that the general public is not aware of and which underlie misjudgments, and valuation difficulties, like focusing and contrast effects, leading the valuations of the general public astray. Arguments to the effect that general public values are more accurate also focus on adaptation issues and valuation difficulties, this time as a particular problem for patient values. Another set of arguments, often referring to ‘social values,’ such as the tax payer/insurance payer argument and the argument about vested interests, uniquely supports general public values.

**Table 1:** Arguments favoring *patients* as the most accurate source for valuing health states (only first author listed, see Table 5, Online Appendix, for full references)

First-level themes	Supporting quotes	Support in literature (citations in italic)
Patients better informed (know better what it is like)	“The argument for using patient values seems to hinge crucially on the fact that patients know the health states better than someone trying to imagine them. … Given the evidence that general population values are poor proxies for those of patients, this implies that patient values should be used.” “If the perspectives of patient and doctor are both valuable for their particular insights, it is less easy to see why this should also be true of the general public. Why should we think the public perspective important, if it lacks both experience and knowledge? It is ironic that current measures rely almost exclusively on eliciting the preferences of the public, when intuitively it seems to be the least authoritative group.”	*Brazier (2005) p. 204* *Wolff (2012) p. 460* Jalukar (1998) Revicki (1998) Gabriel (1999) Nord (1999) De Wit (2000) Ubel (2000) Polsky (2001) Happich (2002) Landy (2002) Menzel (2002) Feeny (2003) Prosser (2003) Stein (2003) Happich (2005) Burström (2006) Rashidi (2006) Ratcliffe (2007) Briggs (2008) Dolan (2008) Lloyd (2008) Stiggelbout (2008) Arnold (2009) Dolan (2009) Gandjour (2010) Garau (2011) Krabbe (2011) McTaggart-Cowan (2011a) McTaggart-Cowan (2011b) Stamuli (2011) McTaggart-Cowan (2012) Rand-Hendriksen (2012) Butt (2013) Burström (2014) Mulhern (2014) Thavorncharoensap (2014) Wang (2014) Whately-Smith (2014) Aronsson (2015) Gandhi (2015) Papageorgiou (2015) Rowen (2015) Schwalm (2015) Dagklis (2016) Jonker (2016) Mott (2016) Versteegh (2016) Gandhi (2017) Sossong (2017)
The general public has an incomplete health state description	“First, the general public does not necessarily know what it is like to experience the specific illnesses being evaluated in CEAs, whereas patients actually experience the illnesses in question. (…) Second, when conducting utility elicitations from the general public, we must describe the health states in question to the public. These descriptions will always be incomplete and, therefore, may introduce bias.”	*Ubel (2000) p 128* Ubel (2000) Happich (2005) Arnold (2009) McTaggart-Cowan (2011a) Schwalm (2015) Sossong (2017)
The general public disregards adaptation	“Patients adapt to their illnesses, whereas people who have not experienced such a health state might fail to anticipate this ability to adapt.”	*Edelaar-Peteers (2012) p. 806* Ubel (2000) Polsky (2001) Menzel (2002) Ubel (2003) Damschroder (2005) Happich (2005) Damschroder (2008) Dolan (2008) Stiggelbout (2008) Gandjour (2010) Garau (2011) McTaggart-Cowan (2011b) McTaggart-Cowan (2012) Wolff (2012) Burström (2014) Sun (2015) Jonker (2016) Versteegh (2016) Rowen (2017)
Activity adjustment (as a form of adaptation)	“Realizing that a disease or disability is likely to be chronic, people may adjust their activities. Still desiring physical exercise, for example, a former cyclist, now paraplegic, may take up aerobic wheelchairing. Or a person may change occupations, not because she has altered her substantive goals in life, but because she now deems a different occupation to be a better avenue for achieving them.”	*Menzel (2002) p. 2151* Versteegh (2016)
Skill enhancement (as a form of adaptation)	“With time, chronically disabled or ill persons may develop greater skill in using whatever physical or mental capacities they retain. No activities or goals are adjusted; people simply improve their ability to accomplish their existing goals in their existing activities, beyond what they previously could ever have imagined was possible.” “…the authors note that ‘skill enhancement,’ ‘activity adjustment’ … could be considered ‘laudable adaptation’…”	*Menzel (2002) p. 2151* *Versteegh (2016) p. 70* McTaggart-Cowan (2011b)
Substantive goal adjustment (as a form of adaptation)	“People may adjust not only the activities they select to pursue their goals, but the content and direction of the goals themselves. Their basic interest has changed.”	*Menzel (2002) p. 2151* Versteegh (2016)
Altered conception of health (as a form of adaptation)	“It is to note that people who have what is commonly thought of as ‘disability’ or ‘disease’ may be stimulated to adopt a radically different and, in their eyes, a more insightful definition of their health.”	*Menzel (2002) p. 2152* McTaggart-Cowan (2011b) Versteegh (2016)
Heightened stoicism (as a form of adaptation)	“They control their happiness so that it is a function only of what they come to see as achievable.”	*Menzel (2002) p. 2152*
Contrast effects (new, forgiving perspective on minor issues)	“For example, if a patient with MS is evaluating the probable quality of life of another hypothetical person with MS, she might have learned from her own experience that MS has made it easier for herself to emotionally deal with minor day-to-day frustrations that used to bother her significantly. (…) A member of the general public, on the other hand, may not consider the likelihood that having MS would create these types of contrast effects.”	*Ubel (2003) p. 604*
Focusing effects (the general public over-emphasize negative aspects of the health state, incl. Peak-start rule and Transition)	“[G]eneral population respondents typically focus on the negative aspects of ill health whilst ignoring unaffected life domains that the descriptive system does not bring to their attention…” “There are at least three factors that tend to inflate the public’s assessments of the severity of hypothetical health states. First … the respondent’s attention is drawn to the transition from one state to another. … Therefore, valuations are likely to be affected by a ‘Peak-Start Rule’ where respondents focus on the worst effects of a health change and the effects that are experienced immediately. For many adverse conditions, the peak and the start will coincide. …"	*Garau (2011) p. 679* *Dolan (2008) p. 71* Ubel (2003) Brazier (2005) McTaggart-Cowan (2011a) Peeters (2011) Stamuli (2011) Wolff (2012) Burström (2014) Wilson (2014) Sun (2015) Versteegh (2016) Leidl (2017)
Reference point (distance renders evaluation more difficult)	“[F]or the general public impaired health may be too hypothetical, causing the general public to overestimate the impact of health impairments yielding rather low, and perhaps relatively often negative, that is "worse than dead", preferences …”	*Versteegh (2016) p. 69*
Valuation compression (distance compresses small differences between health states; the problem from the healthy viewpoint)	“"[T]he general population may undervalue movements between severe states. … [F]or example, at extreme levels of disability the general population may be insensitive to small improvements in mobility that are highly valued by patients.” “Much the way the distance we are from two objects affects our ability to judge the distance between them, people's current health affects their evaluation of severity of other health states.” … “To a person in normal health, the difference between hemiplegia and hemiplegia with aphasia may seem small - both health states are extremely severe. But, to a patient living with either health state, the difference will appear much larger - having or not having the ability to speak makes a big difference to these patients.”	*Brazier (2005) p. 205* *Ubel (2003) p. 602* Damschroder (2005) Garau (2011)
Discrimination avoidance	“'Third, the public may be biased against people with disability or illness, and this may be reflected in value measurements.”	*Ubel (2000) p. 128* Hadorn (1992) Lenert (1999) Brazier (2005)
Protection of the unhealthy	“[T]he general population tend to give a lower health state value than do patients. (…) Giving lower values to the lives of ill people means that life-saving interventions will look less attractive than if patient values had been used…” “The ‘gain’ from an intervention [that is life extending] may then be *higher* when valued by patients” [i.e., when patients value health states higher than the general public].”	*Brazier (2005) p. 205* *Versteegh (2016) p. 70* Ubel (2000) McPherson (2004)
Welfare economics	“An important normative position (or value judgement) in welfare economics is that the well-being of a society is simply the aggregation of the utility of individual members of society. In other words, it asserts the supremacy of an individual’s valuation of their own well-being. This implies that it is the preferences of the losers and gainers from a public programme that should be elicited, and not a sample of the general population who will be unaffected by the change; this would seem to suggest that patient values should be used.” “A strong argument in favor of using patient preferences in the assessment of health states is experience with a disease. What this study adds is a theoretical justification of patient preferences: they can be justified by Harsanyi’s veil-of-ignorance model and, more generally, by preference utilitarianism and welfare economics because their satisfaction increases or maximizes social welfare. … In contrast, this article found no compelling theoretical basis for community preferences for use in QALYs.”	*Brazier (2005) p. 204* *Gandjour (2010) p. E61* De Wit (2000) Burström (2006) Ratcliffe (2007) Brouwer (2008) Lee (2011) Stamuli (2011) Weyler (2011) Wang (2014) Schwalm (2015) Mott (2016) Gries (2016) Leidl (2017)
Patients more affected	“The main rationale for using the patient’s perspective is to place greater emphasis on those most directly affected by a policy.”	*McTaggart-Cowan (2011b), p. 1904* Froberg (1989) Dolan (1996) Gabriel (1999) Neumann (2000) McTaggart-Cowan (2011b)

**Table 2:**
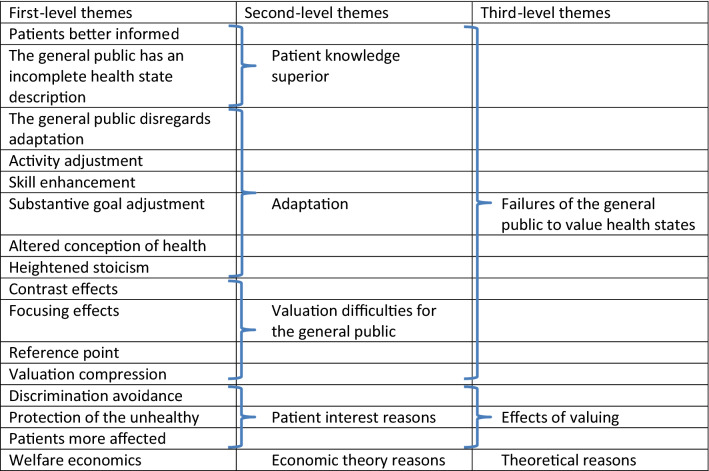
Arguments favoring patients as the most accurate source for valuing health states sorted in themes

**Table 3:** Arguments favoring the *general public* as the most accurate source for valuing health states (only first author listed, see Table 5, Online Appendix, for full references)

First-level themes	Supporting quotes	Support in literature (citations in italic)
Patients overestimate the quality of life of the ill health state (as a form of adaptation)	“Patients’ ability to adapt to illness might mean that they will overestimate the quality of life of specific health conditions.”	*Ubel (2000) p. 130* Rand-Hendriksen (2012) Burström (2014) Ogorevc (2017) Rowen (2017)
Happy slave (as a form of adaptation)	“The ‘happy slave’ example shows why subjective reports of well-being or the expression of someone's preferences might both be inadequate guides to value; even if a slave is happy, and prefers slavery to freedom, nevertheless, it is argued, he or she has real interests that are undermined by slavery." “From an external point of view one can have reason to value some conditions above others even if they are not correlated with greater subjective well-being. As in the 'happy slave' case, the existence of choice, freedom, and opportunity seems to matter.”	*Wolff (2012) pp. 458, 460* Burström (2006)
Cognitive denial (as a form of adaptation)	“Patients may find it difficult to admit how poor their objective, functional health really is. … [I]t hardly seems desirable to base the value of a health state that is used to shape social policy on judgments that are factually mistaken.”	*Menzel (2002) p. 2151* Ubel (2000) McTaggart-Cowan (2011b) Versteegh (2016)
Failure to recognize full health (as a form of adaptation)	“[P]atients cease to realize anymore what full health is like or what it would enable them to do. Here again, it hardly seems desirable for such cognitive blindness to influence the measurement of health state utility…”	*Menzel (2002) p. 2151* Dolan (2008) McTaggart-Cowan (2011b) Jonker (2016) Versteegh (2016) Rowen (2017)
Heightened stoicism	“They [patients] control their happiness so that it is a function only of what they come to see as achievable.”	*Menzel (2002) p. 2152*
Lowered expectations	“Regardless of other, more complex adjustments, chronically ill or disabled persons may simply lower their level of expected achievement, fatalistically accepting their diminished lot in life.” “Some researchers argue that certain elements of adaptation (such as the lowering of expectations) are regrettable and that it would be inappropriate for these factors to influence healthcare prioritization decisions.”	*Mentzel (2002) p. 2152* *Garau (2011) p. 679* Ubel (2000) Ratcliffe (2007) McTaggart-Cowan (2011b) Stamuli (2011) McTaggart-Cowan (2012) Jonker (2016) Versteegh (2016) Rowen (2017)
Response shift	“Imagine a person who is asked before and after the onset of an illness to rate her HRQoL on a 1 to 10 scale. The illness may change her idea of what the numbers 1 and 10 represent, a phenomenon called *response shift* – changes in health lead to changing internal standards for evaluating one’s own health, making it difficult to compare HRQoL before and after illness. (…) Response shift is also related to changing expectations.”	*Ubel (2003) p. 602* Myers (2003) Happich (2005) Finell (2012) McTaggart-Cowan (2012)
Focusing effects due to patients’ recall difficulties (including peak-end-rule)	“There is substantial evidence that people are generally poor at recalling their experiences. People recalling past experiences tend to be subject to a range of biases, such as 'peak-end' effects where they tend to focus on their peak experience and their last experience and, consequently, weight these experiences more heavily than the rest.” “There is now good evidence that the retrospective recall of health is highly correlated with current health state and not so well correlated with the initial state. More generally, our memories do not recall past experiences and their duration particularly well.”	*Brazier (2005) p. 206* *Dolan (2008) p. 72* Ubel (2003)
Reference point (distance render evaluation more difficult)	“The general public is generally healthy and can judge the loss of capabilities from the viewpoint of someone who is in full health. This may result in a 'better', or at least uniform, representation of the 'distance' between being in full health and having the health impairment. For patients the reference point they are reasoning from may have shifted and full health may be too hypothetical, which may be con-sidered problematic when wishing to come to universally applicable health state valuations.”	*Versteegh (2016) p. 69*
Valuation compression (distance compresses small differences between health states; problem from the patient viewpoint)	“When patient values are used in studies aimed at the comparison of different therapeutic modalities for one clinical problem, the ‘valuation compression’ at the upper end of the scale might result in loss of sensitivity to discriminate between the therapeutic modalities, when in fact differences between those modalities exist.”	*de Wit (2000) p. 117*
Societal perspective	“[V]aluations made by the general public may be most appropriate in situations where health care resources are being distributed in the public interest, such as in government funded health systems. Their appropriateness in these instances stems from the belief that valuations made by the general public are the best representation of societal preferences." “[T]he current economic standard is to elicit HRQoL estimates from the general public rather than from patients, because economic analyses are meant to guide social policy and not individual patient decisions.” “[E]ven if the [patients’] values derived would be unbiased in terms of costs, they may not necessarily mirror community preferences, and hence (…) may lack democratic legitimacy.”	*Polsky (2001) p. 34* *Ubel (2003) p. 604* *Happich (2005) p. 51* Neumann (2000) Ubel (2000) Polsky (2001) Happich (2002) Myers (2003) Prosser (2003) McPherson (2004) Brazier (2005) Stein (2005) Ariza-Ariza (2006) Ratcliffe (2007) Dale (2008) Lloyd (2008) Stiggelbout (2008) Arnold (2009) Garau (2011) Lee (2011) McTaggart-Cowan (2011b) Stamuli (2011) Pickard (2013) Burström (2014) Wang (2014) Whately-Smith (2014) Gandhi (2015) Jonker (2016) Mott (2016) Versteegh (2016) Sossong (2017)
Insurance (ex ante)	“Public funding can essentially be seen as public insurance and so it is the *ex ante* public preferences that should be used to value health states.”	*Brazier (2005) p. 204* Ratcliffe (2007) Arnold (2009) Gandjour (2010) Burström (2014) Versteegh (2016)
Tax payer (including insurance payer)	“A point often raised is that in a tax or insurance system of health, the people paying for health care are ordinary citizens, many of whom do not fall seriously ill. It might be claimed that those who pay have a right to determine how their money is spent.” “This decision [based on the Panel on Cost-effectiveness in Health and Medicine, to use values from the general public] was based on the argument that the public bears the costs associated with healthcare decisions, hence they should be part of the decision-making process for the allocation of benefits.”	*Wolff (2012) p. 461* *Stamuli (2011) p. 206* Froberg (1989) Hadorn (1992) Dolan (1996) de Wit (2000) Green (2003) Myers (2003) Brazier (2005) Burström (2006) Rashidi (2006) Ratcliffe (2007) Happich (2009) Gandjour (2010) McTaggart-Cowan (2011a) Rand-Hendriksen (2012) Butt (2013) Burström (2014) Mulhern (2014) Thavorncharoensap (2014) Wang (2014) Whately-Smith (2014) Aronsson (2015) Gandhi (2015) Rowen (2015) Schwalm (2015) Mott (2016) Versteegh (2016) Ogorevc (2017) Rowen (2017)
Welfarism/Extra-welfarism	“It could be argued that every citizen in a public system has an option to use the service and so may be gainers or losers, meaning that the general population would be a good proxy." “… an ‘extra-welfarist’ approach, which considers that any number of stakeholders, such as social decision makers (…) or an average tax-payer, might be regarded as a more appropriate source of values than individual patients.”	*Brazier (2005) p. 204* *Stamuli (2011) p. 205* Arnold (2009) Gries (2016)
Veil of ignorance	“'The Washington Panel went on to use the ‘veil of ignorance’ to support the use of community values, where a ‘rational public decides what is the best course of action when blind to its own self-interest, aggregating the utilities of persons who have not vested interest in particular health states seems most appropriate.’” “[I]t is stated that rational citizens when operating behind a ‘veil of ignorance’, and thus ignorant of their own future health state and needs, would prefer that societal decisions lead to maximum aggregate benefit within that society.”	*Brazier (2005) p. 204* *de Wit (2000) p. 110* Ubel (2000) Happich (2002) Rashidi (2006) Pyne (2009) McTaggart-Cowan (2011a) Gandjour (2010) Stamuli (2011) McTaggart-Cowan (2011b) Rand-Hendriksen (2012) Butt (2013) Thavorncharoensap (2014) Schwalm (2015) Mott (2016) Versteegh (2016) Ogorevc (2017)
Vested interest	“A further argument is that unlike patients, the general population tends not to have a vested interest in getting access to treatment and is, therefore, more likely to give an unbiased view of the value of the health gain it generates.”	*Garau (2011) p. 679* Ashby (1994) Jalukar (1998) Polsky (2001) Happich (2002) Happich (2005) Rashidi (2006) Briggs (2008) Pyne (2009) McTaggart-Cowan (2011a) McTaggart-Cowan (2011b) Stamuli (2011) McTaggart-Cowan (2012) Rand-Hendriksen (2012) Butt (2013) Whately-Smith (2014) Gandhi (2015) Papageorgiou (2015) Rowen (2015) Schwalm (2015) Dagklis (2016) Jonker (2016) Mott (2016) Rowen (2017)
Better for patients that non-adapted health states are considered	“[I]t may be unjust if some patients lost out in the race for resources because their effort diminished the value of treatments for them compared to other patients who did not expend the same effort’. I.e. they have adapted…”	*Dolan (2008) p. 75* Menzel (2002) McPherson (2004)
Better for patients with general public values: the general public rates conditions as worse; gains from treatment hence appear larger	“It has been argued that the use of general population values benefits patients. This is based on the observation that the general population tends to give a lower health state value than do patients. Therefore, for any intervention aimed at curing or preventing a condition associated with ill health states, general population values will generate a larger gain.”	*Brazier (2005) p. 205* Raisch (2000) Versteegh (2016) Ogorevc (2017)
Easier to obtain large amounts of data	“One of the major perceived advantages of such generic measures of health status [letting representatives of the general population evaluate descriptions of health conditions] from the researcher’s perspective lies in their ability to provide ‘off the shelf’ values for a wide variety of generic health states.”	*Ratcliffe (2007) p. 396* Gandjour (2010) Rand-Hendriksen (2012) Wolff (2012) Rowen (2015) Mott (2016) Ogorevc (2017)

**Table 4:**
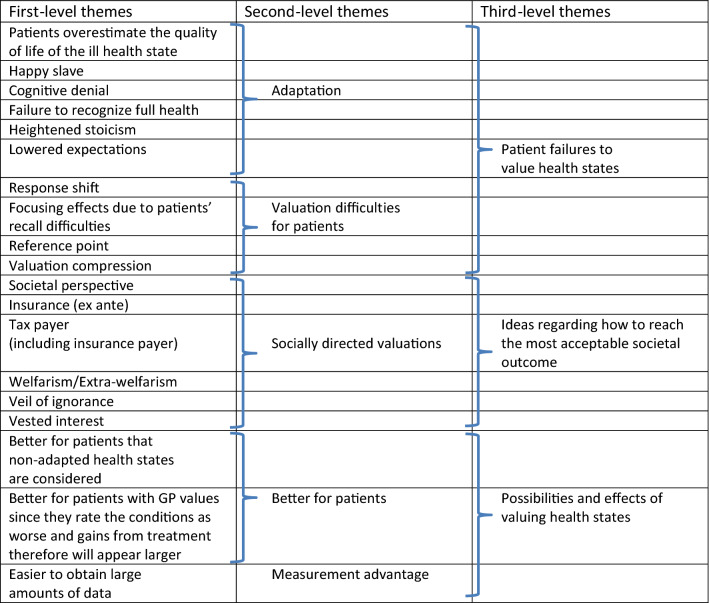
Arguments favoring the general public as the most accurate source for valuing health states sorted in themes

## Discussion

This review of arguments regarding the most accurate source of information for valuation of health states—individuals’ valuations based on their experience of the health state or individuals’ valuations based on a description of the health state, usually framed in the debate as patient values versus general public values—shows that there is an intense discussion in the literature with several arguments for each position (see Tables 1, 3). There is a structural similarity between the two sets of arguments. The arguments supporting the use of valuations made by the general public point out that patients have difficulties making the valuations, partly relating to adaptation [33–35], partly relating to focusing effects and distortions due to perspective (reference point effects), such as that being in a health state very far from full health may lead to difficulties in estimating the distance [2, 3, 16, 36]. This is exactly what the opposing arguments say about the general public, as a reason to favor patient values (although the details of the arguments differ) [3, 16, 36, 37]. In both sets of arguments, there are also arguments to the effect that their position is best when it comes to protecting the interests of patients in need [2, 11, 16, 38]. What distinguishes them is that theoretical reasons based on welfare economics are frequently and explicitly appealed to in support of patient values [2, 21, 30, 39], while arguments relating to social values [36, 40, 41], absence of bias [24, 35, 37, 42], and research advantages [24, 43, 44] are repeatedly brought up in support of using general public values.

### No flawless position

When it comes to evaluating the strength of the two positions, it becomes clear that neither position is flawless. That patients have superior knowledge about the health state they experience, compared to the general public, seems to be unquestionable in many cases. We take this to be the most significant difference between the two approaches. It is argued that the general public also tends to be considerably misled by focusing effects (effects of focusing on some aspects of ill health while not considering unaffected life domains) [3, 42] and to have difficulties understanding the degree to which the individual can adapt to a new health state, even if the change from a healthy state to ill health is dramatic, shocking, and deeply saddening when it occurs [31, 45, 46]. However, there are also convincing arguments that patients in various ways may misrepresent their new situation to themselves. Their own view of what their life is like may be considerably distorted, perhaps because of a need to see it as better than it is [3, 47, 48].

Arguments relating to welfare economics do not put an end to the discussion. As put by Brazier and colleagues [2], p. 204:

An important normative position (or value judgement) in welfare economics is that the well-being of a society is simply the aggregation of the utility of individual members of society. In other words, it asserts the supremacy of an individual’s valuation of their own well-being. This implies that it is the preferences of the losers and gainers from a public programme that should be elicited, and not a sample of the general population who will be unaffected by the change; this would seem to suggest that patient values should be used. One response to this is that current patients do not represent all those likely to be affected by the set of decisions being made. It could be argued that every citizen in a public system has an option to use the service and so may be gainers and losers, meaning that the general population would be a good proxy. However, this presumes that all citizens have an equal chance of receiving all forms of care, which is unlikely. Therefore, welfare economics does not seem to offer a clear argument for either approach.

All this means that there are good reasons not to trust any of the alternatives without reservations. This being said, our review of the arguments found in the published literature suggests that the overall most accurate source of information for valuation of health states is that based on experience (patient values), mainly because those with own experience of a health state are better informed about it. This suggestion is not conclusive, but what would be required of new empirical input to change the balance would be to show that patients as a matter of fact are not better informed about their own health state than those getting the state described to them.

### Irrelevant arguments

One of the most striking features of the debate is that some of the arguments used are entirely irrelevant to the issue at stake here: what is the most accurate source of information for valuation of health states? One example, found in both sets of arguments, is the argument that making the valuation as proposed is advantageous to the patient group [2, 16]. Advantageous or not—this has nothing to do with the question at hand.

A large set of equally irrelevant arguments to the question at hand are found among those supporting the use of the general public valuations of described health states, gathered under the third-level theme ‘ideas regarding how to reach the most acceptable societal outcome.’ The identified first-level themes were referring, for instance, to the societal perspective and tax payer (including insurance payer) arguments. It has been suggested that valuations from the general public for described health states are to be preferred with arguments like “[T]he best articulation of society’s preferences for a particular state would be gathered from a representative sample of fully informed members of the community” [22], “The public, and not patients, should make distributive decisions about who should gain health-related utility as a result of public programmes” (p. 133) [33] and “The social perspective for publicly funded services can also be supported on the grounds that it is tax funded” (p. 204) [2].

Notice, however, that the defense concerns something else than being the most accurate source of information for valuation of the health state; the arguments rather focus on another matter, namely who should exert influence over health policy decisions. There is indeed a fundamental difference between what is the most accurate source of information for valuation of health states and who should get a say regarding policy decisions (which certainly also is worth discussing). As commented by Wolff et al. (p. 461) [26], “Arguably, the political concern about how the public likes to see its money spent should not impinge on the empirical question of how best to measure the benefits of health interventions.” Generally, what is a good answer depends on what is the question. If you want to know the value of a certain health state (which was our question), then that is what you should examine. If you instead want to know how the health state should be handled, what resources should be allocated and how it should be prioritized in relation to other health states in need of attention, then that is what you should investigate. There is no reason to assume beforehand that the answer is the same to both questions. This needs to be argued rather than assumed. Hence, if one believes that prioritization in health care should ultimately be decided by the general population, or should be influenced by their preferences, it still remains to be shown that the general population is also the best source of health state valuations.

This perhaps comes more naturally for those who are not used to understand value in terms of preferences, but rather see it as something supervenient on characteristics of that which is valued. For the latter, the value of a health state is more obviously something distinct from what desires people might have towards that state (i.e., *value of* the health state means something else than *preferences for or proattitudes towards* the health state). Policy-makers might, of course, want several kinds of input, including (i) an accurate valuation of concerned health states and (ii) a description of *perceptions* of different health states among the general public [16].

Arguments about ‘social values’ can also be regarded as irrelevant in another way. People in the debate seem to assume that individuals in the general public generate social values, while patients generate individual values. However, there is nothing more social about the values of individuals from the general public than the values of some patient group. In the words of Brazier et al. (p. 201) [2]:

The conventional approach to valuing health states essentially asks respondents for their ‘off the cuff’ *ex ante* valuation of the states. The resultant values have sometimes been described as social values but this is a misleading term since respondents are being asked to value the states from the perspective of being in the states. The resultant set of health state values is really an average of the individual valuations of those states.

More relevant under this third-level theme, since they do concern the correct valuation of health states, are the veil of ignorance and the vested interests arguments, both stressing the risk that patients valuing their present health states present partisan valuations in order to serve their own interests. In theory, this could present a real problem. However, in practice, these misgivings seem to be unjustified since empirical research shows that patients often provide higher health state values than the general public, which would rather be to their disadvantage in the context of health improving treatments [2].

### Subjective and objective aspects of health states

There are genuine difficulties with the core issue—what should be meant by ‘a health state’? Both subjective and objective considerations might come in here. One way to understand ‘health state’ is that a health state consists of nothing but what it is like to be in that state—health is what it appears to be “from within”—that is, it is an entirely subjective concept. However, if a health state is fully characterized by what it is like to be in that state, then the valuation errors identified in the debate regarding experience-based values—for instance, relating to shifting value scales, focusing effects and reference point effects—are, after all, not errors. More specifically, there will be no external standard (i.e., based outside the patient) by which to label patient valuations as accurate or inaccurate. The obvious implication would be that asking those being in the health state to value it is superior to any other alternative.

However, it can be contested whether a health state exclusively consists of its subjective aspects. For instance, being in a certain health state might restrict the opportunities to do things, a fact stressed both by Norman Daniels [49, 50] and in the discussion of capabilities [8, 26, 51-53]. Whether the individual perceives such limitations as genuine loss is one thing, and whether opportunities are in fact lost, missed or not, is another. One might argue that loss of opportunities or capabilities is a genuine loss whether missed by the individual or not, putting the individual in another health state compared to the state before the loss occurred. Opportunities and capabilities, in other words, bring in an objective (i.e., non-subjective) aspect to the discussion of what constitutes a health state. Only if we allow considerations other than what it is like to be in that state can we question the superiority of experience-based valuations of health states. Although it goes beyond the ambitions of the present study to compare with an opportunity- or capability-based approach, it can be noted that in cases where those being in a certain health state do not see that they have lost anything by ending up in that state, members of the general public may see it because they note opportunities and capabilities lost [26].

So we conclude that if the main problem with using values from the general public for described health states is that they do not know enough about the health states they are valuing and become victims of all kinds of misleading focusing effects, which would most likely remain even if they would receive more information, the main problem for patients as evaluators is that they may be overly focused on what it is like to be in the health state as perceived from their new perspective, while that may not be all there is to say about it.

### Strengths and limitations

The main strength of this paper is that it provides a structured overview of the arguments occurring in the debate regarding most accurate source of information for valuation of health states, and that it identifies some common arguments in the debate as irrelevant for this specific matter. Hopefully it can contribute to an increased focus on the quality of the relevant arguments. We also take it to be a strength of the paper that it is the outcome of collaboration between health economics and ethics.

Although the ambition of this review has been to cover the wide variety of arguments relating to the most accurate source for valuations of health states, patient values or general public values, we cannot exclude that limitations of the search terms or data bases used have made us miss some articles that would have been relevant to our paper. The exclusion of books from the systematic review might have had similar effects, although we suggest that what appears from an author in the form of books often have turned up in print before that in the form of journal papers.

A built-in difficulty with the approach of the present paper is the identification and interpretation of arguments. Many of the arguments identified in the literature are not clearly expressed and quite a few are just briefly sketched rather than thoroughly formulated. Sometimes, it is not clear whether the authors have intended a passage as an argument or if they just point to differences in outcomes depending on the chosen source for health state valuations. It cannot be excluded that we have occasionally failed to identify relevant articles or relevant arguments in the articles reviewed—e.g., because they have been embedded in arguments explicitly concerning something else—or that we have interpreted passages as containing arguments where no argument in favor of the one or the other alternative was intended. It was not part of our review to systematically improve the arguments found in the literature into their best possible version.

Another potential limitation is the organization and structure of the paper. The analysis of arguments has taken its starting point in the existing literature, including the way the topic has been framed, which means that some of the messiness of the discussion, not least the terminological variations, has affected the paper. Furthermore, it is not entirely clear that central terms, like “general public” and “patient values,” are used in the same way by different authors and over time. From an analytical perspective, it might have been more fruitful to use some alternative approach that would have distanced itself more from the existing discourse. But then one would, of course, easily have missed the point of showing what the discussion has looked like. Furthermore, some lack of clarity cannot be circumvented by introducing a new terminology (this would, for instance, not solve potential lack of clarity as to what authors mean by “general public” and “patient values”). The need for clarification and interpretation of the terminology used in health state valuations remains [9, 16, 18]. The extensive search strategy reduced the risk of not finding relevant publications due to the variety in terminology used in health state valuations.

Finally, it cannot be excluded that preconceptions of the authors may have influenced both identification and interpretation of arguments in the debate, although awareness of this risk throughout the work has hopefully tempered such tendencies. The last author has published a paper on experience-based value sets for EQ-5D health states [17].

## Conclusion

This paper has provided a structured overview of the arguments in the discussion in the published literature of the most accurate source of information for valuation of health states—individuals’ valuations based on their experiences of the health state or individuals’ valuations based on a description of the health state. Our review shows that none of these approaches is flawless and that both positions are partly backed up by irrelevant arguments. This is particularly so regarding support for the described health states position, where ideas regarding social values dominate the argumentation while being beside the point for the question of the most accurate source of information for health state valuation, which was the focus of our study. Arguments for both approaches rightly point out that the other approach has difficulties in relation to adaptation: the general public valuing described states underestimates the ability of those in ill health to adapt, while those valuing the health state based on their experience may have adapted in ways that distort their valuation. Both approaches also have difficulties relating to focusing and reference point effects. Nevertheless, our review of the argumentation in the published literature suggests that the most accurate source of information for valuation of health states is that based on experience, mainly because those with own experience of the health state are better informed about it. This suggestion is not conclusive, but what is required of new empirical input to change the balance is to show that those with experience of the state are as a matter of fact not better informed about their own health state than those getting the state described to them.

## Electronic supplementary material

Below is the link to the electronic supplementary material.

Supplementary material (DOCX 23 kb)

Supplementary material (DOCX 31 kb)

## References

[1] Nord E (1999). Cost-value analysis in health care.

[2] Brazier J, Akehurst R, Brennan A (2005). Should patients have a greater role in valuing health states?. Applied Health Economics and Health Policy.

[3] Dolan P (2008). Developing methods that really do value the ‘Q’ in the QALY. Health Economics, Policy and Law.

[4] Dolan P, Kahneman D (2008). Interpretations of utility and their implications for the valuation of health. The Economic Journal.

[5] Dolan P (2009). NICE should value real experiences over hypothetical opinions. Nature.

[6] Drummond M, Brixner D, Gold M (2009). Toward a consensus on the QALY. Value in Health.

[7] Drummond MF, Sculpher MJ, Claxton K, Stoddart GL, Torrance GW (2015). Methods for the economic evaluation of health care programmes.

[8] Hausman DM (2015). Valuing health. Well-being, freedom, and suffering.

[9] Brazier J, Rowen D, Karimi M (2018). Experienced-based utility and own health state valuation for a health state classification system: Why and how to do it. The European Journal of Health Economics.

[10] Brazier J, Ratcliffe J, Saloman J, Tsuchiya A (2016). Measuring and valuing health benefits for economic evaluation.

[11] McPherson K, Myers J, Taylor WJ, McNaughton HK, Weatherall M (2004). Self-valuation and societal valuations of health state differ with disease severity in chronic and disabling conditions. Medical Care.

[12] Mann R, Brazier J, Tsuchiya A (2009). A comparison of patient and general population weightings of the EQ-5D dimensions. Health Economics.

[13] Burström K, Johannesson M, Diderichsen F (2006). A comparison of individual and social time trade-off values for health states in the general population. Health Policy.

[14] Krabbe PFM, Tromp N, Ruers TJM, van Riel PLCM (2011). Are patients’ judgements of health status really different from the general population?. Health and Quality of Life Outcomes.

[15] McNamee P (2007). What difference does it make? The calculation of QALY gains from health profiles using patient and general population values. Health Policy.

[16] Versteegh MM, Brouwer WBF (2016). Patient and general public preferences for health states: A call to reconsider current guidelines. Social Science and Medicine.

[17] Burström K, Sun S, Gerdtham UG (2014). Swedish experience-based value sets for EQ-5D health states. Quality of Life Research.

[18] Cubi-Molla P, Shah K, Burström K (2018). Experience-based values: A framework for classifying different types of experience in health valuation research. Patient.

[19] Leidl R, Reitmeir P (2011). A value set for the EQ-5D based on experienced health states Development and testing for the German population. Pharmacoeconomics.

[20] Sun S, Chen J, Kind P (2015). Experience-based VAS values for EQ-5D-3L health states in a national general population health survey in China. Quality of Life Research.

[21] Leidl R, Reitmeir P (2017). An experience-based value set for the EQ-5D-5L in Germany. Value in Health.

[22] Gold MR, Siegel JE, Russell LB, Weinstein MC (1996). Cost-effectiveness in health and medicine.

[23] Kind, P. (2009). Valuing EQ-5D health states—A VAStly simpler solution? In J. Busschbach, R. Rabin, F. de Charro (Eds.), *Proceedings of the 24th scientific plenary meeting of the EuroQol Group* (pp. 319–337). Kijkduin-The Hague, The Netherlands Rotterdam: EuroQol Group Executive Office. Retrieved 9 September 2015.

[24] Rand-Hendriksen K, Augestad LA, Kristiansen IS, Stavem K (2012). Comparison of hypothetical and experienced EQ-5D valuations: Relative weights of the five dimensions. Quality of Life Research.

[25] Leidl R, Reitmeir P, König HH, Stark R (2012). The performance of a value set for the EQ-5D based on experienced health states in patients with inflammatory bowel disease. Value in Health.

[26] Wolff J, Edwards S, Richmond S, Orr S, Rees G (2012). Evaluating interventions in health: A reconciliatory approach. Bioethics.

[27] National Institute for Health and Care Excellence (NICE). (2013). Guide to the methods of technology appraisal 2013. London: National Institute for Health and Care Excellence: London. Retrieved 26 June, 2018, from https://www.nice.org.uk/process/pmg9/chapter/foreword. 27905712

[28] The Dental and Pharmaceutical Benefits Agency (TLV). (2003). General guidelines for economic evaluations from the Pharmaceutical Benefits Board (LFNAR 2003:2). Stockholm: The Dental and Pharmaceutical Benefits Agency. Retrieved 26 June, 2018, from https://www.tlv.se/download/18.2e53241415e842ce95514e9/1510316396792/Guidelines-for-economic-evaluations-LFNAR-2003-2.pdf.

[29] The Dental and Pharmaceutical Benefits Agency (TLV). (2017). General guidelines for economic evaluations from the Dental and Pharmaceutical Benefits Agency (TLVAR 2017:1). Stockholm: The Dental and Pharmaceutical Benefits Agency. Retrieved 26 June, 2018, from https://www.tlv.se/download/18.467926b615d084471ac3230c/1510316374332/TLVAR_2017_1.pdf.

[30] Brouwer WBF, Culyer AJ, van Exel NJA, Rutten FFH (2008). Welfarism vs. extra-welfarism. Journal of Health Economics.

[31] Sandelowski M (2000). Whatever happened to qualitative description?. Research in Nursing & Health.

[32] Graneheim UH, Lundman B (2004). Qualitative content analysis in nursing research: Concepts, procedures and measures to achieve trustworthiness. Nurse Education Today.

[33] Ubel PA, Nord E, Gold M, Menzel P, Prades JL, Richardson J (2000). Improving value measurement in cost-effectiveness analysis. Medical Care.

[34] Ogorevc M, Murovec N, Fernandez NB, Rupel VP (2017). Questioning the differences between general public vs. patient based preferences towards EQ-5D-5L defined hypothetical health states. Health Policy.

[35] Rowen D, Zouraq IA, Chevrou-Severac H, van Hout B (2017). International regulations and recommendations for utility data for health technology assessment. PharmacoEconomics.

[36] Ubel PA, Loewenstein G, Jepson C (2003). Whose quality of life? A commentary exploring discrepancies between health state evaluations of patients and the general public. Quality of Life Research.

[37] Stamuli E (2011). Health outcomes in economic evaluation: who should value health?. British Medical Bulletin.

[38] Lenert LA, Treadwell JR, Schwartz CE (1999). Associations between health status and utilities implications for policy. Medical Care.

[39] de Wit GA, Busschbach JJV, de Charro FTH (2000). Sensitivity and perspective in the valuation of health status: whose values count?. Health Economics.

[40] Polsky D, Willke RJ, Scott K, Schulman KA, Glick HA (2001). A comparison of scoring weights for the EUROQOL derived from patients and the general public. Health Economics.

[41] Happich M, von Lengerke T (2005). Valuing the health state ‘tinnitus’: differences between patients and the general public. Hearing Research.

[42] Garau M, Shah KK, Mason AR (2011). Using QALYs in cancer A review of the methodological limitations. Pharmacoeconomics.

[43] Ratcliffe J, Brazier J, Palfreyman S, Michaels J (2007). A comparison of patient and population values for health states in varicose veins patients. Health Economics.

[44] Gandjour A (2010). Theoretical foundation of patient v population preferences in calculating QALYs. Medical Decision Making.

[45] Menzel P, Dolan P, Richardson J, Olsen JA (2002). The role of adaptation to disability and disease in health state valuation: A preliminary normative analysis. Social Science and Medicine.

[46] Edelaar-Peeters Y, Putter H, Snoek GJ (2012). The influence of time and adaptation on health state valuations in patients with spinal cord injury. Medical Decision Making.

[47] Myers JA, McPherson KM, Taylor WJ, Weatherall M, McNaughton HK (2003). Duration of condition is unrelated to health-state valuation on the EuroQol. Clinical Rehabilitation.

[48] McTaggart-Cowan HM, O’Cathain A, Tsuchiya A, Brazier JE (2012). Using mixed methods research to explore the effect of an adaptation exercise on general population valuations of health states. Quality of Life Research.

[49] Daniels N (1985). Just health care.

[50] Daniels N (2007). Just health.

[51] Sen A (1985). Commodities and capabilities.

[52] Sen A (1992). Inequality reexamined.

[53] Nussbaum M, Sen A (1993). The quality of life.

