# Supplementary material for: Whom should we ask? A systematic literature review of the arguments regarding the most accurate source of information for valuation of health states
Source: Qual Life Res. 2020 Feb 3;29(6):1465–82. doi: 10.1007/s11136-020-02426-4 (PMC7253527; doi:10.1007/s11136-020-02426-4)
Supplement: Supplementary file 1 — Supplementary material (DOCX 23 kb) [file 11136_2020_2426_MOESM1_ESM.docx]

Helgesson G, et al. Whom should we ask? *Quality of Life Research*. Appendix A.

## Search strategies for Medline, Scopus and Econlit

The original search in Medline, Scopus, and Econlit was performed October 12, 2015. A complementary search was performed November 1, 2017. The searches within respective database were identical apart from the difference in time restrictions. The tables below show the search strategies.

| **No.** | **Search in Medline** |
| --- | --- |
| 1 | ((health state or health states) adj5 (valu* or evaluat* or preference*)).tw. |
| 2 | (preference* adj3 elicit*).tw. |
| 3 | (utilit* or qaly* or quality-adjusted life year*).tw. |
| 4 | Quality-Adjusted Life Years.sh. |
| 5 | Or/1-4 |
| 6 | (experienc* or patient or patients or individual or individuals).tw. |
| 7 | (ex adj post).tw. |
| 8 | Or/6-7 |
| 9 | Hypothetical.tw. |
| 10 | ((social or societal or community or population or public) adj (valu* or tariff* or preference*)).tw. |
| 11 | (general adj (public or population)).tw. |
| 12 | (ex adj ante).tw. |
| 13 | Or/9-12 |
| 14 | 5 and 8 and 13 |

.tw. = title/abstract
adj3 = adjacent 3 words regardless of order
adj5 = adjacent 5 words regardless of order
adj = adjacent word in specified order
.sh. = MeSH term

In the complementary search, the time restriction was set to the years: 2015-current (i.e., Nov. 1, 2017)

| **No.** | **Search in Scopus*** |
| --- | --- |
| 1 | Title-abs((“health state” OR “health states”) W/5 (valu* OR evaluat* OR preference*)) |
| 2 | Title-abs(preference* W/3 elicit*) |
| 3 | Title-abs(utilit* OR qaly* OR quality-adjusted life year*) |
| 4 | Or/1-3 |
| 5 | Title-abs(experienc* OR patient OR patients OR individual OR individuals) |
| 6 | Title-abs(ex PRE/0 post) |
| 7 | Or/5-6 |
| 8 | Title-abs(hypothetical) |
| 9 | Title-abs((social OR societal OR community OR population OR public) PRE/0 (valu* OR tariff* OR preference*)) |
| 10 | Title-abs(general PRE/0 population) OR (general PRE/0 public) |
| 11 | Title-abs(ex PRE/0 ante) |
| 12 | Or/8-11 |
| 13 | 4 and 7 and 12 ^1^ |

Title-abs = title/abstract
W/3 = adjacent within 3 words regardless of order
W/5 = adjacent within 5 words regardless of order
PRE/0 = adjacent word in specified order
*Limited to scientific journals

In the complementary search, the time restriction was set to the years: 2015-2018

| **No.** | **Search in Econlit*** |
| --- | --- |
| 1 | AB((“health state” OR “health states”) AND (valu* OR evaluat* OR preference*)) |
| 2 | AB(preference* AND elicit*) |
| 3 | AB(utilit* OR qaly* OR quality-adjusted life year*) |
| 4 | Or/1-3 |
| 5 | AB(experienc* OR patient OR patients OR individual OR individuals) |
| 6 | AB “ex post” |
| 7 | Or/5-6 |
| 8 | AB hypothetical |
| 9 | AB(social OR societal OR community OR population OR public) AND (valu* OR evaluat* OR preference*) |
| 10 | AB(“general public” OR “general population”) |
| 11 | AB “ex ante” |
| 12 | Or/8-11 |
| 13 | 4 and 7 and 12 |

AB = abstract
* limited to: academic journals

In the complementary search, the time restriction was set to the years: 2015-2017
