# Supplementary material for: Whom should we ask? A systematic literature review of the arguments regarding the most accurate source of information for valuation of health states
Source: Qual Life Res. 2020 Feb 3;29(6):1465–82. doi: 10.1007/s11136-020-02426-4 (PMC7253527; doi:10.1007/s11136-020-02426-4)
Supplement: Supplementary file 2 — Supplementary material (DOCX 31 kb) [file 11136_2020_2426_MOESM2_ESM.docx]

Table 5. Articles listed in tables 1 and 3

Ariza-Ariza R, Hernandez-Cruz B, Carmona L et al. Assessing utility values in rheumatoid arthritis: A comparison between time trade-off and the EuroQol. Arthritis Rheum 2006;55:5:751-756.

Arnold D, Girling A, Stevens A, Lilford R. Comparison of direct and indirect methods of estimating health state utilities for resource allocation: review and empirical analysis. BMJ 2009;339.

Aronsson M, Husberg M, Kalkan A, Eckard N, Alwin J. Differences between hypothetical and experience-based value sets for EQ-5D used in Sweden: Implications for decision makers. Scand J Public Health 2015;43:848-854.

Ashby J, O’Hanlon M, Buxton MJ. The time trade-off technique: how do the valuations of breast cancer patients compare to those of other groups? Qual Life Res 1994;3:257-265.

Brazier J, Akehurst R, Brennan A, et al. Should patients have a greater role in valuing health states? Appl Health Econ Health Policy 2005; 4:4:201-208.

Briggs A, Wild D, Lees M et al. Impact of schizophrenia and schizophrenia treatment-related adverse events on quality of life: direct utility elicitation. Health Qual Life Outcomes 2008;6;105.

Brouwer WBF, Culyer AJ, van Exel NJA, Rutten FFH. Welfarism vs. extra-welfarism. J Health Econ 2008;27:325-338.

Burström K, Johannesson M, Diderichsen F. A comparison of individual and social time trade-off values for health states in the general population. Health Policy 2006;76:359-370.

Burström K, Sun S, Gerdtham UG et al. Swedish experience-based value sets for EQ-5D health states. Qual Life Res 2014;23:431-442.

Butt T, Dunbar HMP, Morris S, Orr S, Rubin GS. Patient and public preferences for health states associated with AMD. Optom Vis Sci 2013;90:8:855-860.

Dagklis IE, Aletras VH, Tsantaki E, Orologas A, Niakas D. Multiple sclerosis patients valuing their own health status: valuation and psychometric properties of the 15D. Neurol Int 2016;8:6416:42-48.

Dale PL, Hutton J, Elgazzar H. Utility of health states in chronic kidney disease: a structured review of the literature. Curr Med Res Opin 24;1:193-206.

Damschroder L J, Zikmund-Fisher BJ, Ubel PA. The impact of considering adaptation in health state valuation. Soc Sci Med 2005;61:267-277.

Damschroder L J, Zikmund-Fisher BJ, Ubel PA. Considering adaptation in preference elicitations. Health Psychology 2008;27:3:394-399.

Dolan P. The effect of experience of illness on health state valuations. J Clin Epidemiol 1996;49:5:551-564.

Dolan P. Developing methods that really do value the ‘Q’ in the QALY. Health Econ Policy Law 2008;3:1:69-77.

Dolan P. NICE should value real experiences over hypothetical opinions. Nature 2009;462:7269:35.

Edelaar-Peeters Y, Putter H, Snoek GJ, et al. The influence of time and adaptation on health state valuations in patients with spinal cord injury. Med Decis Making 2012;32:805-814.

Feeny D, Blanchard C, Mahon JL et al. Comparing community-preference-based and direct standard gamble utility scores: Evidence from elective total hip arthroplasty. Int J Technol Assess Health Care 2003;19:2:362-372.

Finnell SME, Carroll AE, Downs SM. Application of classic utilities to published pediatric cost-utility studies. Acad Pediatr 2012;12:219-228.

Froberg DG, Kane RL. Methodology for measuring health-state preferences-III: population and context effects. J Clin Epidemiol 1989;42:6:585-592.

Gabriel SE, Kneeland TS, Melton L J et al. Health-related quality of life in economic evaluations for osteoporosis: Whose values should we use? Med Decis Making 1999;19:141-148.

Gandhi M, Thumboo J, Luo N, Wee HL, Cheung YB. Do chronic disease patients value generic health states differently from individuals with no chronic disease? A case of a multicultural Asian population. Health Qual Life Outcomes 2015;13:8.

Gandhi M, Tan RS, Ng R, et al. Comparison of health state values derived from patients and individuals from the general population. Qual Life Res 2017;26(12):3353-3363.

Gandjour A. Theoretical foundation of patient v. population preferences in calculating QALYs. Med Decis Making 2010;30:57-63.

Garau M, Shah KK, Mason AR, et al. Using QALYs in cancer. A review of the methodological limitations. Pharmacosecon 2011;29:8:673-685.

Green C, Kiebert G, Murphy C, et al. Patients’ health-related quality-of-life and health state values for motor neurone disease/amyotrophic lateral sclerosis. Qual Life Res 2003;12:565-574.

Gries KS, Regier DA, Ramsey SD, Patrick DL. Preferences for prostate cancer outcomes: A comparison of the patient perspective, the general population perspective, and a population at risk for prostate cancer. Value Health 2016;19:218-225.

Hadorn, DC. The problem of discrimination in health care priority setting. JAMA 1992;268:11:1454-9.

Happich M, Mazurek B. Priorities and prospect theory. Eur J Health Econom 2002;3:40-46.

Happich M, von Lengerke T. Valuing the health state ‘tinnitus’: differences between patients and the general public. Hear Res 2005;207:50-58.

Happich M, Moock J, von Lengerke T. Health state valuation methods and reference points: the case of tinnitus. Value Health 2009;12:1:88-95.

Jalukar V, Funk GF, Christensen AJ, Karnell LH, Moran PJ. Health states following head and neck cancer treatment: patient, health-care professional, and public perspectives. Head Neck 1998;10:600-608.

Jonker MF, Attema AE, Donkers B, Stolk, EA, Versteegh MM. Are health state valuations from the general public biased? A test of health state reference dependency using self-assessed health and an efficient discrete choice experiment. Health Econ 2017;26:12:1534-1547.

Krabbe PFM, Tromp N, Ruers TJM, van Riel PLCM. Are patients’ judgements of health status really different from the general population? Health Qual Life Outcomes 2011;9:31.

Landy J, Stein JD, Brown MM, Brown GC, Sharma S. Patient, community and clinician perceptions of the quality of life associated with diabetes mellitus. Med Sci Monit 2002;8:8:543-548.

Lee JM, Rhee K, O’Grady MJ et al. Health utilities for children and adults with Type 1 diabetes. Med Care 2011;49(10) 924-931.

Leidl R, Reitmeir P. An experience-based value set for the EQ-5D-5L in Germany. Value Health 2017;20:1150-1156.

Lenert LA, Treadwell JR, Schwartz CE. Associations between health status and utilities implications for policy. Med Care 1999;37:5:479-489.

Lloyd A, van Hanswijck de Jonge P, Doyle S, Cornes P. Health state utility scores for cancer-related anemia through societal and patient valuations. Value Health 2008;11:7:1178-1185.

McPherson K, Myers J, Taylor WJ, McNaughton HK, Weatherall M. Self-valuation and societal valuations of health state differ with disease severity in chronic and disabling conditions. Med Care 2004;42:11:1143-1151.

McTaggart-Cowan H, Tsuchiya A, O’Cathain A, Brazier J. Understanding the effect of disease adaptation information on general population values for hypothetical health states. Soc Sci Med 2011;72:1904-1912.

McTaggart-Cowan H. Elicitation of informed general population health state utility values: a review of the literature. Value Health 2011;14:1153-1157.

McTaggart-Cowan HM, O’Cathain A, Tsuchiya A, Brazier JE. Using mixed methods research to explore the effect of an adaptation exercise on general population valuations of health states. Qual Life Res 2012;21:465-473.

Menzel P, Dolan P, Richardson J, Olsen JA. The role of adaptation to disability and disease in health state valuation: a preliminary normative analysis. Soc Sci Med 2002;55:2149-2158.

Mott DJ, Najafzadeh M. Whose preferences should be elicited for use in health-care decision-making? A case study using anticoagulant therapy. Expert Rev Pharmacoecon Outcomes Res 2016;16:1:33-39.

Mulhern B, Rowen D, Snape D, et al. Valuations of epilepsy-specific health states: a comparison of patients with epilepsy and the general population. Epilepsy Behav 2014;36:12-17.

Myers JA, McPherson KM, Taylor WJ, Weatherall M, McNaughton HK. Duration of condition is unrelated to health-state valuation on the EuroQol. Clin Rehab 2003;17:209-215.

Neumann PJ, Goldie SJ, Weinstein MC. Preference-based measures in economic evaluation in health care. Annu Rev Public Health 2000;21:587-611.

Nord E, Pinto JL, Richardson J, Menzel P, Ubel P. Incorporating societal concerns for fairness in numerical valuations of health programmes. Health Econ 1999;8:25-39.

Ogorevc M, Murovec N, Fernandez NB, Rupel VP. Questioning the differences between general public vs. patient based preferences towards EQ-5D-5L defined hypothetical health states. Health Policy 2017, Mar 28. doi: 10.1016/j.healthpol.2017.03.011

Papageorgiou K, Vermeulen KM, Schroevers MJ, et al. Do individuals with and without depression value depression differently? And if so, why? Qual Life Res 2015;24:2565-2575.

Peeters Y. Vlieland TPMV, Stiggelbout AM. Focusing illusion, adaptation and EQ-5D health state descriptions: the difference between patients and public. Health Expect 2011;15:367-378.

Pickard AS, Tawk R, Shaw JW. The effect of chronic conditions on stated preferences for health. Eur J Health Econ 2013;14:697-702.

Polsky D, Willke RJ, Scott K, Schulman KA, Glick HA. A comparison of scoring weights for the EUROQOL derived from patients and the general public. Health Econ 2001;10:27-37.

Prosser LA, Kuntz KM, Bar-Or A, Weinstein MC. Patient and community preferences for treatments and health states in multiple sclerosis. Multiple Sclerosis 2003;9:311-319.

Pyne JM, Fortney JC, Tripathi S, et al. How bad is depression? Preference score estimates from depressed patients and the general population. Health Services Res 2009;44:4:1406-1423.

Raisch DW. Understanding quality-adjusted life years and their application to pharmacoeconomic research. Ann Pharmacother 2000;34.

Rand-Hendriksen K, Augestad LA, Kristiansen IS, Stavem K. Comparison of hypothetical and experienced EQ-5D valuations: relative weights of the five dimensions. Qual Life Res 2012;21:1005-1012.

Rashidi AA, Anis AH, Marra CA. Do visual analogue scale (VAS) derived standard gamble (SG) utilities agree with Health Utilities Index utilities? A comparison of patient and community preferences for health status in rheumatoid arthritis patients. Health Qual Life Outcomes 2006;4:25.

Ratcliffe J, Brazier J, Palfreyman S, Michaels J. A comparison of patient and population values for health states in varicose veins patients. Health Econ 2007;16:395-405.

Revicki DA, Wood M. Patient-assigned health state utilities for depression-related outcomes: differences by depression severity and antidepressant medications. J Affect Disord 1998;48:25-36.

Rowen D, Mulhern B, Banerjee S et al. Comparison of general population, patient, and carer utility values for dementia health states. Med Decis Making 2015;35:1:68-80.

Rowen D, Zouraq IA, Chevrou-Severac H, van Hout B. International regulations and recommendations for utility data for health technology assessment. Pharmacoecon 2017;35(Suppl 1):11-19.

Schwalm A, Feng YS, Moock J, Kohlmann T. Differences in EQ-5D-3L health state valuations among patients with musculoskeletal diseases, health care professionals and healthy volunteers. Eur J Health Econ 2015;16:865-877.

Sossong B, Felder S, Wolff M, Krüger K. Evaluating the consequences of rheumatoid arthritis. Eur J Health Econ 2017;18:685-696.

Stamuli E. Health outcomes in economic evaluation: who should value health? Br Med Bull 2011;97:197-210.

Stein JD, Brown MM, Brown GC, Hollands H, Sharma S. Quality of life with macular degeneration: perceptions of patients, clinicians, and community members. Br J Ophthalmol 2003;87:8-12.

Stein K, Fry A, Round A, Milne R, Brazier J. What value health? A review of health state values used in early technology assessments for NICE. Appl Health Econ Health Policy 2005;4(suppl 4):219-228.

Stiggelbout AM, de Vogel-Voogt E. Health state utilities: a framework for studying the gap between the imagined and the real. Value Health 2008;11:1:76-87.

Sun S, Chen J, Kind P et al. Experience-based VAS values for EQ-5D-3L health states in a national general population health survey in China. Qual Life Res 2015;24:693-703.

Thavorncharoensap M. Measurement of utility. J Med Assoc Thai 2014; 97 (Suppl 5):43-49.

Ubel PA, Nord E, Gold M et al. Improving value measurement in cost-effectiveness analysis. Med Care 2000;38:9:892-901.

Ubel PA, Richardson J, Menzel P. Societal value, the person trade-off and the dilemma of whose values to measure for cost-effectiveness analysis. Health Econ 2000;9:127-136.

Ubel PA, Loewenstein G, Jepson C. Whose quality of life? A commentary exploring discrepancies between health state evaluations of patients and the general public. Qual Life Res 2003;12:599-607.

Versteegh MM, Brouwer WBF. Patient and general public preferences for health states: A call to reconsider current guidelines. Soc Sci Med 2016;165:66-74.

Wang P, Tai ES, Thumboo J, Vrijhoef HJM, Luo N. Does diabetes have an impact on health-state utility? A study of Asians in Singapore. Patient 2014;7:329-337.

Weyler EJ, Gandjour A. Empirical validation of patient versus population preferences in calculating QALYs. Health Serv Res 2011;46(5):1562-1574.

Whately-Smith C, Watkins C, Mann H, Fletcher C, Ducournau P. Utility values in health technology assessments: a statistician’s perspective. Pharmaceut Statist 2014;13:184-195.

Wilson R, Hansen P, Langley J, Derrett S. A comparison of injured patient and general population valuations of EQ-5D health states for New Zealand. Health Qual Life Outcomes 2014;12:21.

de Wit GA, Busschbach JJV, de Charro FTH. Sensitivity and perspective in the valuation of health status: whose values count? Health Econ 2000; 9:109-126.

Wolff J, Edwards S, Richmond S, Orr S, Rees G. Evaluating interventions in health: a reconciliatory approach. Bioethics 2012;26:9:455-463.
